# Common Variants in *PLXNA4* and Correlation to CSF-related Phenotypes in Alzheimer's Disease

**DOI:** 10.3389/fnins.2018.00946

**Published:** 2018-12-18

**Authors:** Qiu Han, Yong-An Sun, Yu Zong, Chun Chen, Hui-Fu Wang, Lan Tan, Alzheimer's Disease Neuroimaging Initiative

**Affiliations:** ^1^Department of Neurology, Qingdao Clinical Medical School, Qingdao Municipal Hospital, Nanjing Medical University, Qingdao, China; ^2^Department of Neurology, The Affiliated Huaian Hosipital of Xuzhou Medical University, Huai'an, China; ^3^Department of Neurology, First Affiliated Hospital of Kangda School, Nanjing Medical University, Lianyungang, China; ^4^Department of Neurology, School of Medicine, Qingdao Municipal Hospital, Qingdao University, Qingdao, China; ^5^Department of Neurology, Hongze Huai'an District People's Hospital, Huai'an, China

**Keywords:** Plexin-A 4 (*PLXNA4*), variant, Alzheimer's disease (AD), amyloid-β (Aβ), association

## Abstract

The Plexin-A 4 (*PLXNA4*) gene, has recently been identified in genome wide association studies (GWAS), as a novel genetic player associated with Alzheimer's disease (AD). Additionally, *PLXNA4* genetic variations were also found to increase AD risk by tau pathology *in vitro*. However, the potential roles of *PLXNA4* variants in the amyloid-β (Aβ) pathology, were not evaluated. Five targeted loci capturing the top common variations in *PLXNA4*, were extracted using tagger methods. Multiple linear regression models were used to explore whether these variations can affect the cerebrospinal fluid (CSF) (Aβ_1−42_, T-tau, and P-tau) phenotypes in the Alzheimer's disease Neuroimaging Initiative (ADNI) dataset. We detected that two loci (rs6467431, rs67468325) were significantly associated with CSF Aβ_1−42_ levels in the hybrid population (rs6467431: *P* = 0.01376, rs67468325: *P* = 0.006536) and the significance remained after false discovery rate (FDR) correction (rs6467431: *P*c = 0.03441, rs67468325: *P*c = 0.03268). In the subgroup analysis, we further confirmed the association of rs6467431 in the cognitively normal (CN) subgroup (*P* = 0.01904, *P*c = 0.04761). Furthermore, rs6467431-A carriers and rs67468325-G carriers showed higher CSF Aβ_1−42_ levels than non-carriers. Nevertheless, we did not detect any significant relationships between the levels of T-tau, P-tau and these *PLXNA4* loci. Our findings provided preliminary evidence that *PLXNA4* variants can confer AD risk through modulating the Aβ deposition.

## Introduction

Globally, an estimated 47 million people had dementia in 2015 and this figure is projected to triple by 2050 (Livingston et al., 2017). AD is the leading cause of dementia and is one of the biggest health-care challenges of the twenty first century (Scheltens et al., 2016). The earliest recognizable pathological event in AD is brain amyloid-β aggregation (Bateman et al., 2012). As the initial event and the most important factor, it is responsible for a battery of downstream abnormalities in AD (Cummings et al., 2007; Mormino et al., 2009; Jack et al., 2010, 2013). Novel research criteria for AD in the preclinical phase, highlight the occurrence of amyloid pathology in the first stage of the disease (Sperling et al., 2011; Dubois et al., 2014). However, the etiological mechanisms underlying the neuropathological changes remain unclear (Reitz and Mayeux, 2014). Family studies revealed that complex genetic mechanisms along with environmental factors, contribute to disease risk (Jiang et al., 2013; Yu et al., 2014). Indeed, the estimated heritability for late-onset Alzheimer's disease (LOAD) is between 60 and 80% (Gatz et al., 2006), suggesting that genetic determinants are involved in most of the pathophysiological pathways in AD. Therefore, genetics might play an important role in the underlying pathogenesis of AD.

Plexin-A 4, a member of the Plexin-A family, which is located on chromosome 7q32.3, is a receptor for secreted semaphorin class 3 (SEMA3A) and class 6 (SEMA6) proteins, which mediate the effects of multiple semaphorins, including controlling diverse aspects of the nervous system development and plasticity, ranging from axon guidance and neuron migration to synaptic organization (Sun et al., 2013; Andermatt et al., 2014; Kong et al., 2016). Previous studies have shown that Plexin-A 4 (encoded by *PLXNA4*) is activated by Sema3A and can mediate growth cone collapse and axon repulsion, as well as dendrite morphogenesis in different neuronal populations during development (Suto et al., 2005; Yaron et al., 2005; Tran et al., 2009; Pasterkamp, 2012), both in cells and *in vivo*. Accumulation of SEMA3A was previously detected in susceptible areas of the hippocampal neurons during AD progression and colocalized with phosphorylated tau (Good et al., 2004). Phosphorylated CRMP2 protein, an intracellular signaling molecule for the semaphorin-plexin signaling pathway, has been observed in neurofibrillary tangles in the brains of autopsied AD patients (Cole et al., 2004).

A genome-wide significant association study (GWAS) on humans has identified that several single nucleotide polymorphisms (SNPs) in *PLXNA4* can increase the risk of AD (Jun et al., 2014). However, the concrete mechanisms *PLXNA4* contribute to AD risk, remain elusive. Jun et al. found that tau phosphorylation mediated by *PLXNA4* is an independent upstream event contributing to AD-related neurofibrillary tangles in neurons *in vitro* (Jun et al., 2014). Additionally, Kang et al. demonstrated that Plexin A4 (*PLXNA4*) is a novel, high affinity receptor for *CLU* in the adult CNS (Kang et al., 2016). Their data demonstrated that the level of *PLXNA4* significantly impacts CSF levels of *CLU in vivo* in mice and that *PLXNA4* is genetically associated with *CLU* levels in the CSF of humans (Kang et al., 2016). Nevertheless, induction of intracellular signal pathways mediated by *PLXNA4* in the adult brain in general, or particularly by *CLU-PLXNA4* interactions, remain unexplored (Kang et al., 2016). Cogent evidence reveals that the physiological interplay of *CLU* with Aβ has an important influence on AD pathogenesis (Yu and Tan, 2012). We therefore hypothesized that *PLXNA4* would also modulate Aβ accumulation to modify AD risk, compared to tau pathology.

By demonstrating that these genetic risk factors of AD also influence CSF traits, important verification of the roles for these loci will be supported and will indicate the mechanisms by which they might act. To test this hypothesis, we aimed to explore the involvement of *PLXNA4* variants in the underlying pathogenesis of AD, by investigating the effects of *PLXNA4* polymorphisms on the brain amyloid burden through CSF phenotypes in the ADNI dataset.

## Materials and Methods

### ADNI Database

The ADNI is a large, multicentered, ongoing, longitudinal neuroimaging study, started in 2003 by the National Institute on Aging, the National Institute of Biomedical Imaging and Bioengineering, the Food and Drug Administration, nonprofit organizations, and private pharmaceutical companies (Mueller et al., 2005). As an initial goal, 800 subjects were recruited, but the ADNI has since been followed by ADNI-GO and ADNI-2. Thus far, over 1,500 adults (aged between 55 and 90) have been recruited in the three protocols, including people with AD, people with early or late MCI and cognitively normal older individuals. This study was upheld by the institutional review boards of centers involved and written informed consent was acquired from all participants or from authorized representatives, after a comprehensive description of the ADNI was provided, in accordance with the 1975 Declaration of Helsinki (Carrillo et al., 2012). The present study was authorized by the institutional review boards of Qingdao Municipal Hospital, Memory, and Aging Center at the University of California, and the ADNI. Informed consent was acquired from all participants or from authorized representatives. Additionally, we conformed to the approved guidelines when performing these methods.

### Participants

We included all participants from the ADNI database (http://adni.loni.usc.edu), in this study. Based on the National Institute of Neurological and Communication Disorders/Alzheimer's Disease and Related Disorders Association criteria for probable AD (NINCDS/ADRDA: probable AD), AD patients were enrolled if they had a Mini Mental State Examination (MMSE) score of between 20 and 26, a CDRSB score of between 1.0 and 9.0 and a global Clinical Dementia Rating (CDR) score of between 0.5 and 1.0. Amnestic MCI cases had a MMSE score of between 24 and 30 and a CDR score of at least 0.5, while cognitively normal individuals had a CDR score of 0. Moreover, individuals were excluded if they had a history of brain lesions or trauma, psychoactive medication use (including chronic anxiolytics, antidepressants, sedative hypnotics, or neuroleptics), or serious neurological diseases other than possible AD. Finally, the present study included 812 subjects, including 48 AD, 483 MCI, and 281 CN individuals at baseline. We downloaded the basic data of individuals in our study from the ADNI website in 2015.

### Genotyping and SNP Selection

We used Bead Studio 3.2 software and a recent Genome Studio v2009.1 (Illumina) to produce SNP genotypes from bead intensity data (Saykin et al., 2010). In this study, we extracted the *PLXNA4* genotypes in the ADNI PLINK data format and conducted the quality control procedures using PLINK software. We filtered criteria as follows: minimum minor allele frequencies (MAF) >0.01, Hardy-Weinberg equilibrium test *P* > 0.001, minimum call rates >90%. We preferentially chose eight SNPs (rs277470, rs277472, rs277484, rs277476, rs12539196, rs75460865, rs13232207, rs10273901) reported to be significantly associated with AD for analysis (Jun et al., 2014). Furthermore, five promising tag SNPs (rs78036292, rs6467431, rs67468325, rs1863015, rs156676), capturing the top common variations in *PLXNA4* were chosen with tagger methods in the Haploview 4.2 platform. A total of 13 SNPs were initially screened and eight reported SNPs were excluded due to their absence in the ADNI. Ultimately, the remaining five loci were selected as the target SNPs (Table 2) in our study.

### CSF Biomarkers

Firstly, CSF specimens were collected and transported to the ADNI Biomarker Core laboratory at the University of Pennsylvania Medical Center, within dry ice and a preparation of aliquots (0.5 ml) was prepared from the collected specimens after thawing (1 h) at room temperature and moderate mixing, then preserved in barcode–labeled polypropylene vials in −80°C. The CSF biomarkers, such as Aβ_1−42_, Phosphorylated tau181p and Total-tau, were detected via the multiplex xMAP Luminex platform (Luminex Corp, Austin, TX) using Innogenetics (INNO-BIA AlzBio3; Ghent, Belgium; for research use-only reagents) immunoassay kit–based reagents. Quality control procedures and further analysis details are displayed on the website (http://adni.loni.ucla.edu). This study was a cross-sectional evaluation at baseline with regard to CSF measures. Finally, a total of 627 cases (206 CN, 377 MCI, and 44 AD) with a baseline CSF and corresponding genetic data, were included in the CSF analysis from the ADNI database.

### Statistical Analyses

Differences in continuous variables were compared with the one-way analysis of variance (ANOVA) and categorical variables were examined with a chi-square test. Possible correlations between various biomarkers and the *PLXNA4* genotypes were tested with a multiple linear regression model which controlled gender, education, age, and the *APOE4* status. FDR, a statistical method developed by Hochberg and Benjamini (Hochberg and Benjamini, 1990), was used to adjust multiple hypothesis testing instead of a Bonferroni correction, which was inappropriate due to the non-independence of tests (Biffi et al., 2010). *P* < 0.05 was defined as a significant difference according to the FDR correction. Firstly, we screened significant CSF-related phenotypes associated with *PLXNA4* loci in all individuals. We then stratified subjects into three groups (CN, MCI, and AD) to explore the influences of the *PLXNA4* variants in these phenotypes at deferent clinical stages separately. All statistical analyses were conducted by PLINK 1.07 (http://pngu.mgh.harvard.edu/wpurcell/plink/) and R 3.12 (http://www.r-project.org/).

## Results

### Baseline Characteristics of Included Subjects

The baseline information of individuals included in the study are summarized in Table 1. Finally, 48 AD patients (18 women, 75.51 ± 9.23 years), 483 MCI (201 women, 72.28 ± 7.45 years), and 281 cognitively normal individuals (145 women, 74.51 ± 5.56 years) were enrolled in our study (Table 1). Compared to the MCI and CN group, patients in the AD group had the highest frequency of the ε4 allele within the *APOE* gene as well as the lowest cognitive function based on the scores of the five neuropsychological scales (ADAS11, MMSE, ADAS13, RAVLT, FAQ), lower CSF Aβ_1−42_ levels, higher CSF T-tau and P-tau levels, and the most severe atrophy in the hippocampus.

**Table 1:** The characteristics of the ADNI subjects at baseline.

**characteristics**	***n***	**CN**	***n***	**MCI**	**n**	**AD**	***P***
Age (years)	281	74.51 ± 5.56	483	72.28 ± 7.45	48	75.51 ± 9.23	<0.01
Gender (male/female)	281	136/145	483	282/201	48	30/18	0.035
Education (years)	281	16.41 ± 2.66	483	15.98 ± 2.82	48	15.73 ± 2.62	0.079
APOE ε4 (0/1/2)	281	204/70/7	483	262/180/41	48	14/25/9	<0.01
CDR-SB	207	0.03 ± 0.13	406	1.44 ± 0.87	47	4.44 ± 1.69	<0.01
MMSE	281	29.07 ± 1.15	483	27.89 ± 1.69	48	22.96 ± 2.03	<0.01
ADAS-cog	281	9.06 ± 4.23	480	15.30 ± 6.65	48	29.80 ± 8.44	<0.01
RAVLT	280	44.83 ± 9.60	483	36.16 ± 10.86	47	22.32 ± 7.84	<0.01
FAQ	281	0.17 ± 0.66	481	2.85 ± 3.99	48	12.6 ± 7.14	<0.01
Hippocampus (mm^3^)	257	*7, 344*±895	422	*6, 996*±1126	39	*5, 757*±948	<0.01
CSF Aβ_1−42_ (pg/ml)	206	200.4 ± 52.86	377	175.02 ± 51.71	44	140.5 ± 41.77	<0.01
CSF T-tau (pg/ml)	205	70.1 ± 32.51	374	83.56 ± 48.01	42	124.38 ± 53.85	<0.01
CSF P-tau (pg/ml)	206	30.92 ± 14.84	377	39.31 ± 23.27	44	59.4 ± 31.36	<0.01

*CN, cognitively normal; MCI, mild cognition impairment; AD, Alzheimer's disease; CDR-SB, Clinical Dementia Rating sum of boxes; MMSE, Mini-Mental State Exam; ADAS-cog, Alzheimer's disease Assessment Scale Cognition; RAVLT, Rey Auditory Verbal Learning Test; FAQ, Functional Activities Questionnaire. Data are given as proportions for categorical variables and means ± SD for continuous variables. p-values were assessed with analyses of a chi-square tests or a one-way analysis of variance (ANOVA)*.

**Table 2:** The characteristics of targeted SNP.

**SNP**	**Chr**	**Position**	**Allele change**	**MAF**	**H-W(*p*-value)**
rs78036292	7	intron variant	T→ G	0.0172	0.1635
rs6467431	7	intron variant	G→ A	0.3884	0.7747
rs67468325	7	intron variant	A→ G	0.0543	0.5127
rs1863015	7	intron variant	A→ G	0.4681	0.5225
rs156676	7	intron variant	G→ A	0.2644	0.0295

*SNP, single nucleotide polymorphism; Chr, chromosome; MAF, minimum minor allele frequencies; H-W, Hardy-Weinberg equilibrium test*.

### Genotypes of *PLXNA4* Effect on CSF Markers

**Figure 1:**
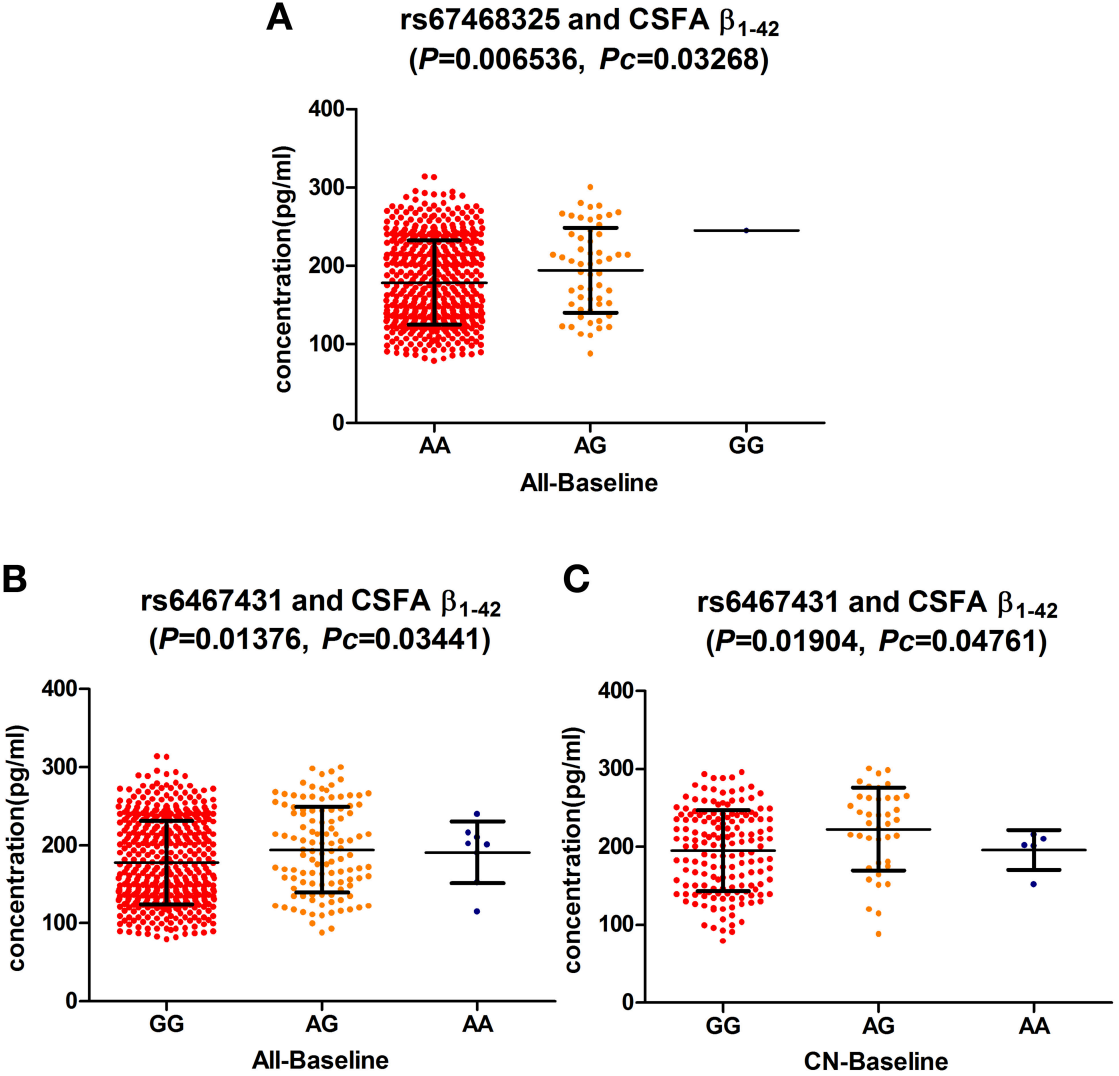
**(A)** The significant association of *PLXNA4* rs67468325 with Cerebrospinal fluid (CSF) Aβ1-42 levels in the hybrid population. (**B)** The significant association of *PLXNA4* rs6467431 with Cerebrospinal fluid (CSF) Aβ1-42 levels in the hybrid population. **(C)** The significant association of *PLXNA4* rs6467431 with Cerebrospinal fluid (CSF) Aβ1-42 levels in the CN group.

At the baseline, we found that two loci (rs6467431, rs67468325) showed marked associations with the levels of CSF Aβ_1−42_, in the hybrid group (rs6467431: *P* = 0.01376, rs67468325: *P* = 0.006536; Figure 1) and the difference reached the significance level in the FDR test (rs6467431: *P*c = 0.03441; rs67468325: *P*c = 0.03268; Figure 1). For rs6467431, minor A allele carriers had higher CSF Aβ_1−42_ levels than non-carriers (AG>AA>GG). For rs67468325, minor G allele carriers had higher CSF Aβ_1−42_ levels in a dose-dependent manner (GG>AG>AA). The minor allele carriers had higher CSF Aβ_1−42_ levels, suggesting that the minor A allele for rs6467431 and G allele for rs67468325, were correlated with less amyloid loads. To determine this result, we then conducted a subgroup analyses and only confirmed the association between the levels of Aβ and rs6467431 in the CN subgroup (*P* = 0.01904, *P*c = 0.04761; Figure 1). In the CN subgroup, rs6467431-A carriers had higher CSF Aβ_1−42_ levels than G allele homozygotes subjects (AG>AA>GG). Although rs67468325 showed a trend to associate with less amyloid loads in the MCI subgroup (*P* = 0.0443), it failed in the FDR test (*P*c = 0.2215). Nevertheless, we did not find any remarkable relationships between the levels of T-tau, P-tau and these *PLXNA4* loci in any individuals and any subgroups.

## Discussion

To our knowledge, this is the first study to explore the roles of *PLXNA4* SNPs in AD pathogenesis using CSF methods. Our main findings indicated that two *PLXNA4* variants modified the risk for AD through Aβ pathology. Genotypes at the *PLXNA4* loci (rs6467431, rs67468325) were related to the trajectory of the Aβ deposition, assuming minor allele effects and these results were significant independent of the *APOE* status. Nevertheless, *PLXNA4* genotypes did not affect CSF tau levels.

Jun et al. conducted a two-stage family-based AD GWAS, using a novel method which incorporates the entire family structure and reduces diagnostic misclassification in the association test and renders the result less prone to type I errors, even for rare variants (Choi et al., 2011; Jun et al., 2014). The analysis of genotyped SNPs (*n* = 341, 492 post quality control) in the Framingham Heart Study (FHS) dataset, using the normalized liability scores for AD, indicated little genomic inflation (λ = 1.01) with strong evidence of an association in three regions of the genome (Jun et al., 2014). A genome-wide significant association was identified with a *PLXNA4* locus (rs277470; meta-analysis *p*-value [meta-*P*] = 4.1 × 10^−8^; Jun et al., 2014). Moreover, these *PLXNA4* loci (rs277470, rs277472, rs277476, and rs277484) were also significant (*P* < 10^−9^ for each) and in complete linkage disequilibrium (LD) (Jun et al., 2014). The most significant SNPs (rs10273901 in Caucasians [meta-*P*] = 3.9 × 10^−5^, rs75460865 in African Americans [meta-*P*] = 8.0 × 10^−4^, rs13232207 in Japanese *P* = 1.2 × 10^−4^) in each population group, were further examined with the meta-analyzed results in the Alzheimer's Disease Genetics Consortium **(**ADGC) datasets (Jun et al., 2014). The results support the association of *PLXNA4* SNPs with AD in these ethnic samples in the ADGC datasets. It was regrettable that we could not extract these *PLXNA4* SNPs due to the absence of these variants in the ADNI database. We therefore extracted 5 targeted *PLXNA4* loci to explore their relations with CSF markers in the ADNI dataset using tagger methods. Our CSF-related Phenotypes analysis in the ADNI dataset showed that *PLXNA4* (rs6467431, rs67468325) were strongly associated with CSF Aβ_1−42_ levels in all subjects. We then validated the association between the levels of CSF Aβ_1−42_ and rs6467431 in the CN subgroup, suggesting that rs6467431 may act in the preclinical phase. MAF for rs6467431 almost 40%, a common variant in *PLXNA4*rs6467431 mainly correlated with Aβ deposition and minor A allele carriers had higher CSF Aβ_1−42_ levels than G allele homozygotes subjects in the CN subgroup (AG>AA>GG). All the above results showed good agreement with previous reports that amyloid pathology often occurs in older CN individuals, at the early stage of the disease (Aizenstein et al., 2008; Jack et al., 2010, 2013; Sperling et al., 2011). Furthermore, rs6467431-A carriers and rs67468325-G carriers had higher CSF Aβ_1−42_ levels than non-carriers, suggesting that the minor A and G allele play protective roles in AD, respectively.

Thus far, few investigations have provided biological evidence of the influence of *PLXNA4* on AD. A vitro study revealed that *PLXNA4* plays a role in AD pathogenesis through isoform-specific influences on tau phosphorylation (Jun et al., 2014). Intriguingly, no evidence supports that *PLXNA4* genotypes affect CSF tau levels, in our study. There are several reasons that might be responsible for this discrepancy. Firstly, a lack of power arose from the limited effect size of the individual variations. Secondly, abnormal levels of tau appear later than the occurrence of the Aβ deposition (Jack et al., 2010, 2013; Buchhave et al., 2012) and we could not observe longitudinal changes of CSF tau due to cross-sectional measurements. Furthermore, as experimental research *in vitro*, Jun's findings could not completely reflect the effects of *PLXNA4* on tau phosphorylation under physiological circumstances. In physiological conditions, the targeted genetic variants can perform a direct effect on phenotypes, but also mediate an influence through the interaction with other genes, or via a downstream functional change. Future studies should ascertain whether these SNPs are authentic regulatory variants (and if so, how it may impact protein expression) or if they are simply flagging a region comprised of another functional variant (Roussotte et al., 2015).

Genetic studies with endophenotypes, not only support enough power to identify novel relations with smaller sample sizes than case–control studies, but can also assist in understanding biological mechanisms of disease (Deming et al., 2017). However, some caveats in this study should be noted. Firstly, our sample was of limited size especially in the subgroup. Secondly, our participants were limited to Caucasians, to prevent genetic stratification across ethnicities and our findings can therefore not be generalized to other ethnicities. Thirdly, we could not obtain longitudinal changes of CSF biomarkers due to the cross-sectional nature of the present study. Furthermore, these *PLXNA4* loci were restricted because of a lack of exact replications. These results should therefore be treated with caution and future replication studies with larger sample sizes, different ethnicities, and longer follow-up are imperative to validate these findings.

## Conclusions

In summary, we detected that two *PLXNA4* loci (rs6467431, rs67468325) correlated with amyloid loads, suggesting that *PLXNA4* may participate in pathogenesis of AD through Aβ pathology. These findings further support that *PLXNA4* genotypes modulate the alterations of Aβ deposition to modify the susceptibility of AD. Although these variants mainly impact subjects at the baseline, it could still provide clues to the underlying relationship between *PLXNA4* and AD. Further studies are warranted to unravel the detailed mechanisms underlying the impacts of *PLXNA4* on AD.

## Author Contributions

LT designed the study Y-AS and QH analyzed the data QH drafted the manuscript LT and QH revised the manuscript QH, H-FW, CC, Y-AS, YZ, and LT interpreted the findings of study. All authors approved the final version for publication.

## Alzheimer's Disease Neuroimaging Initiative

Data used in preparation of this article were obtained from the Alzheimer's Disease Neuroimaging Initiative (ADNI) database (http://adni.loni.usc.edu). As such, the investigators within the ADNI contributed to the design and implementation of ADNI and/or provided data but did not participate in analysis or writing of this report. A complete listing of ADNI investigators can be found at: http://adni.loni.usc.edu/wp-content/uploads/how_to_apply/ADNI_Acknowledgement_List.pdf.

### Conflict of Interest Statement

The authors declare that the research was conducted in the absence of any commercial or financial relationships that could be construed as a potential conflict of interest.
